# A Temperature Control Method for Microaccelerometer Chips Based on Genetic Algorithm and Fuzzy PID Control

**DOI:** 10.3390/mi12121511

**Published:** 2021-12-04

**Authors:** Jiaxiao Chen, Qianbo Lu, Jian Bai, Xiang Xu, Yuan Yao, Weidong Fang

**Affiliations:** 1 State Key Laboratory of Modern Optical Instrumentation, Zhejiang University, Hangzhou 310027, China; jiaxiaochen@zju.edu.cn (J.C.); iconyang@zju.edu.cn (X.X.); fangwd@zju.edu.cn (W.F.); 2 Frontiers Science Center for Flexible Electronics (FSCFE), MIIT Key Laboratory of Flexible Electronics (KLoFE), Shaanxi Key Laboratory of Flexible Electronics (KLoFE), Institute of Flexible Electronics (IFE), Ningbo Institute of Northwestern Polytechnical University, Northwestern Polytechnical University, Xi’an 710072, China; 3 Huazhong Institute of Electro-Optics-Wuhan National Lab for Optoelectronics, Wuhan 430074, China; opt_yaoyuan@126.com

**Keywords:** accelerometers, temperature control, fuzzy logic, genetic algorithms, microelectromechanical devices

## Abstract

External temperature changes can detrimentally affect the properties of a microaccelerometer, especially for high-precision accelerometers. Temperature control is the fundamental method to reduce the thermal effect on microaccelerometer chips, although high-performance control has remained elusive using the conventional proportional-integral-derivative (PID) control method. This paper proposes a modified approach based on a genetic algorithm and fuzzy PID, which yields a profound improvement compared with the typical PID method. A sandwiched microaccelerometer chip with a measurement resistor and a heating resistor on the substrate serves as the hardware object, and the transfer function is identified by a self-built measurement system. The initial parameters of the modified PID are obtained through the genetic algorithm, whereas a fuzzy strategy is implemented to enable real-time adjustment. According to the simulation results, the proposed temperature control method has the advantages of a fast response, short settling time, small overshoot, small steady-state error, and strong robustness. It outperforms the normal PID method and previously reported counterparts. This design method as well as the approach can be of practical use and applied to chip-level package structures.

## 1. Introduction

Accelerometers are widely used in various fields, such as inertial navigation and earthquake monitoring. Microaccelerometers have the advantages of small size, low cost, and high integration [1]. Microaccelerometers can be classified into micro-electromechanical system (MEMS) accelerometers and micro-opto-electromechanical system (MOEMS) accelerometers. MOEMS accelerometers combined with optical measurement technology incorporate anti-electromagnetic interference and higher sensitivity than MEMS accelerometers [2,3]. MOEMS accelerometers based on a grating interferometric cavity have been verified to gain high sensitivity and resolution [4,5], but in practice, they are susceptible to external influences, in which temperature is one of the main sources of error [6]. Microaccelerometers are usually made of silicon and other materials, whose expansion coefficients and elastic modulus are susceptible to ambient temperature changes [7,8]. The performance aspects of MOEMS accelerometers, such as resolution and stability, are more prone to ambient temperature drift because of their extremely high sensitivity. Therefore, it is necessary to precisely control the temperature of the microaccelerometer chip to obtain a constant temperature condition and enhance the chip’s performance.

There are three approaches to reduce the thermal effect on microaccelerometers. The first is structure optimization [9,10,11,12], the second is temperature compensation [13,14,15,16,17,18], and the third is using a temperature control system [19,20]. Structure optimization improves the temperature stability, but it will increase the process complexity and prolong the processing time, and also this method is more suitable for the initial phase of the design. Temperature compensation is relatively simple in structure, but it requires extensive prior experimentation and suffers from machining error. A temperature control system can fundamentally address the temperature effect issue by controlling the temperature inside the microaccelerometer chip, at the expense of complexity. For example, Lee et al. [19] integrated a microheater on the cantilever beam of a piezoresistive MEMS accelerometer for temperature control, and Lakdawala et al. [20] integrated polysilicon heaters embedded within a micromachined sensor structure to maintain the sensor at a constant temperature. These studies can be used as a reference but cannot be directly applied to our system because of the difference in chip design. There are other studies of temperature control methods that work for microsensors. Xia et al. [21] used an integral-separated proportional-integral-derivative (PID) control algorithm in a microgyroscope temperature control system. Xu et al. [22] presented a novel gyroscope prototype with an on-chip heater and temperature sensor. Yang et al. [23] presented the on-chip temperature compensation and temperature control research for the silicon microgyroscope and used a PI (proportional-integral) algorithm for control. Xia et al. [24] presented a temperature control system of microgyroscope based on fuzzy logic controller and the increment PID control method. Chen et al. [25] applied to gyroscope temperature control system from a self-adaptive ANN (artificial neural network) and adopted an adaptive optimization PID algorithm. Jiang et al. [26] introduced a temperature control system that uses the PID control algorithm optimized by the ant colony algorithm in MEMS gyroscopes. These studies gave ideas to use on-chip integrated heaters and PID control methods, but the temperature stability is not high enough for our application. It is worthwhile to systematically investigate a high-performance and widely applicable temperature control approach for microaccelerometer chips, especially for those with high sensitivity because structure optimization and temperature compensation would inevitably bring about side effects.

Our temperature control method has a special application scenario, wherein the output of the micro-optic accelerometer is ultimately sensitive to the variation of the cavity length; hence, it requires extremely high stability and ultrasmall overshoot for temperature control. The control method in this paper is based on the genetic algorithm and fuzzy PID control. Fuzzy logic control can make the parameters adaptively change in the temperature control process. Based on fuzzy PID, we combined it with the genetic algorithm to optimize the best combination of PID control parameters by designing our fitness function. This method has universal applicability and can be applied to the case where there are special requirements for certain parametric quantities. The multiobjective optimization can also be performed to include multiple performance indicators within the fitness function to achieve the best combination of PID control parameters.

In this paper, we give a complete scheme of a temperature control method for a microaccelerometer chip. The hardware object is a semi-closed structure in which the measurement resistor and the heating resistor are integrated, which is shown in Section 2. The detailed control model and control method including fuzzy logic and the genetic algorithm are presented in Section 3. The parameters of the controlled plant are identified by conducting an open-loop step response experiment, and the PID control parameters are modified by fuzzy control and a genetic algorithm to enhance performance, including for robustness and stability. Simulation results of the temperature control are presented in Section 4 to demonstrate that the method has faster response and better steady-state characteristics, which holds the promise of practical application in microsensing chips.

## 2. Controlled Hardware Object

**Figure 1:**
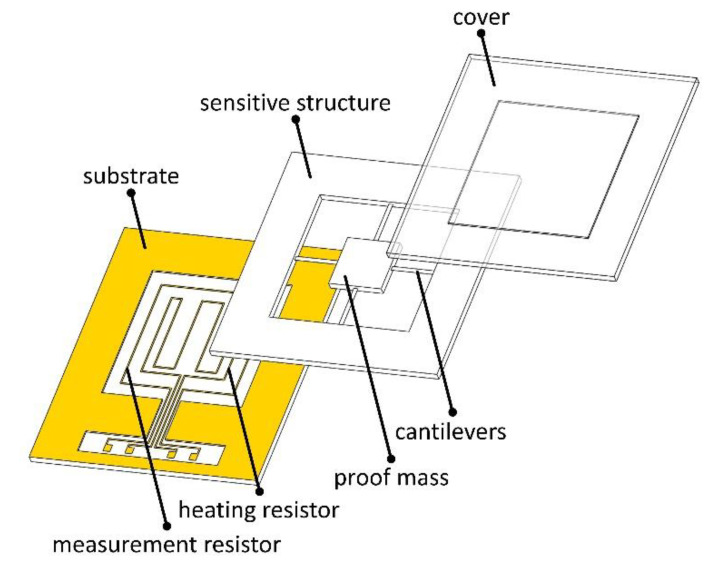
Controlled hardware object.

The controlled hardware object in this paper is a semiclosed microaccelerometer chip. The chip consists of three layers, as shown in Figure 1, namely, the cover, the sensitive structure, and the substrate from top to bottom, whose materials are glass, silicon, and silicon, respectively. The three layers stick together by bonding, forming a cavity, which facilitates the control of the temperature inside the chip and can also effectively protect the sensitive structure. The sensitive structure layer contains a proof mass and cantilevers. Resistors include a measurement resistor and a heating resistor, which are made of platinum through sputtering and etching on a silicon substrate [6]. The pads on the chip are connected to the larger pads on the PCB (printed circuit board) via gold leads. The resistance of the measurement resistor was set to 430 ohms, and the heating resistor was set to 625 ohms at room temperature.

The sandwich-type package can isolate the environmental impact, but it still suffers from external temperature changes due to the coefficient of thermal expansion of the material and the bonding material. Previous experiments showed that the displacement thermal drift of the discrete prototype was 619.54 ± 2.95 nm/°C [6]. This leads to a significant accuracy degradation of the accelerometer output, so controlling the chip’s temperature is necessary.

Resistors on the substrate are used to control the internal temperature of the structure. The resistivity of the platinum metal changes with temperature, and the thermal characteristics of the platinum resistance in the range 0–850 °C can be obtained from the IEC60751:2008 standard:

(1) $$R_{T}=R_{0}\left(1+AT+BT^{2}\right),$$

in which *T* denotes the temperature in Celsius, *R_T_* denotes the resistance at temperature *T*, *R*_0_ denotes the resistance at *T* = 0 °C, and *A* and *B* are constant parameters.

**Figure 2:**
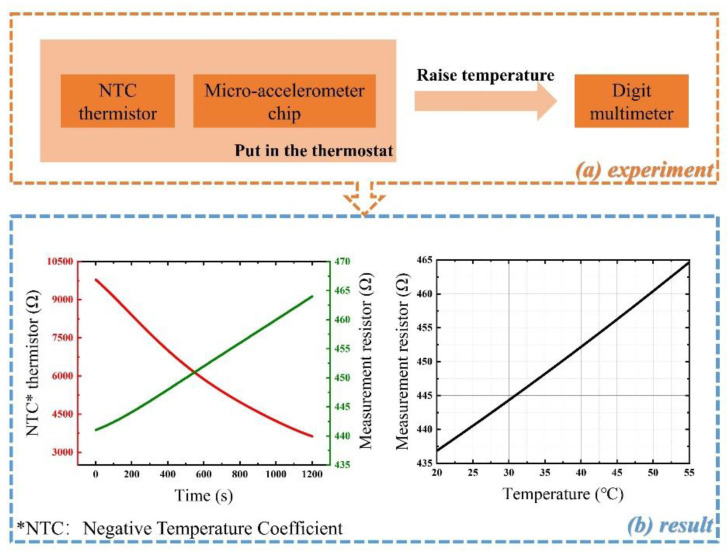
Measurement resistor calibration experiment flow chart.

The relationship between the resistance value of the measurement resistor and the temperature can be roughly obtained through the experiment illustrated in Figure 2. We placed a standard thermistor close to the microaccelerometer chip and put them into the thermostat together and then measured the resistance of the standard thermistor and the measurement resistor simultaneously while the temperature rise. The experimental setup is shown at the top of Figure 2. During the test, a standard NTC (negative temperature coefficient) thermistor was employed as a datum because the thermostat was not accurate enough. The temperature value and the resistance of the measurement resistor were fitted with Equation (1), and the results are shown at the bottom of Figure 2.

## 3. Temperature Control Method Design

In this section, the temperature control model will be determined as PID control, and the least-squares method will be used for parameter identification of the transfer function of the controlled plant. In addition, PID control parameters are adaptively adjusted with fuzzy control and optimized by the genetic algorithm.

### 3.1. Selection of Temperature Control Model

This paper utilizes a negative feedback system based on PID control, which takes the output value as the feedback and takes a linear combination of proportional, integral and differential of error as the controller output. The error is calculated as follows:

(2) e(t)=r(t)−y(t),

in which *e*(*t*), *r*(*t*), and *y*(*t*) denote the error, target value, and output value at a specific point of time *t*, respectively.

Incremental PID does not require accumulation in the calculation process, and the control increment is only related to the last three sampled values, which is more robust to system failures. Herein, we choose incremental PID control in terms of its merit of the figure, and it can be described in a discrete form by the following equations:

(3) $$\Delta u(t)=K_{p}\times \left[e\left(t\right)-e\left(t-1\right)\right]+K_{i}\times T\times e\left(t\right)+\frac{K_{d}}{T}\times \left[e\left(t\right)-2e\left(t-1\right)+e\left(t-2\right)\right],$$

(4) u(t)=u(t−1)+Δu(t),

in which *u*(*t*) denotes the controller output at moment *t*, *e*(*t*) denotes the error at moment *t*, *T* denotes the sampling period, and *K_p_*, *K_i_*, and *K_d_* denote the proportional coefficient, the integration coefficient, and the differential coefficient, respectively.

**Figure 3:**
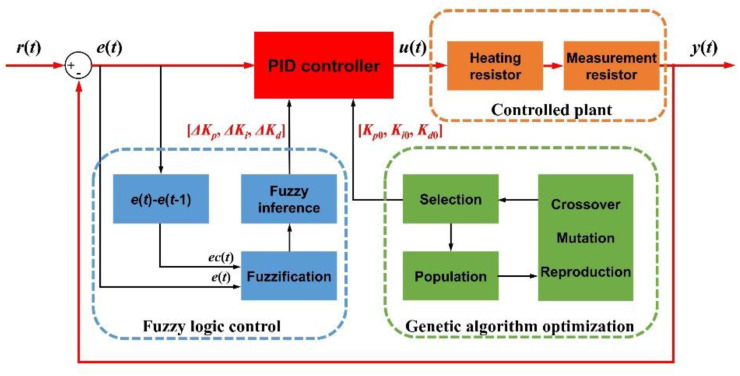
Framework of temperature control methods.

The flow of the temperature control model is shown in Figure 3. *r*(*t*) is the target value at moment *t*, which is the input of the temperature control system, while *y*(*t*) is the output. *e*(*t*) is the input of the PID controller, while the output *u*(*t*) acts on the controlled plant, including the heating resistor and the measurement resistor. The transfer function of the controlled plant should be identified by a step response experiment. The parameters of the PID controller are adaptively adjusted by applying fuzzy logic control. Generally, *e*(*t*) is the error at moment *t* and *ec*(*t*) = *e*(*t*) − *e*(*t* − 1) is the change of error at moment *t*, both of which are used as the inputs of the fuzzy logic control, and the adjustment parameters [Δ*K_p_*, Δ*K_i_*, Δ*K_d_*] of the PID controller are output after fuzzification and fuzzy inference so that control parameters of PID will change continuously during the adjustment process to improve the control performance. The initial parameters [*K_p_*_0_, *K_i_*_0_, *K_d_*_0_] of the PID controller are obtained by genetic algorithm optimization, and the optimal combination of parameters is selected according to the fitness function through crossover, mutation, and reproduction of the population to obtain optimal temperature control results. The final time-varying PID control parameters [*K_p_*(*t*), *K_i_*(*t*), *K_d_*(*t*)] are shown as:

(5) $$\{\begin{array}{c} K_{p}\left(t\right)=K_{p0}+\Delta K_{p}\\ K_{i}\left(t\right)=K_{i0}+\Delta K_{i}\\ K_{d}\left(t\right)=K_{d0}+\Delta K_{d} \end{array}.$$

### 3.2. Identification of the Transfer Funcion of the Controlled Plant

In the PID temperature control system, the transfer function of the controlled plant needs to be identified. In this paper, the heating resistor and the measurement resistor on the substrate layer are the controlled plant in the system. The heating resistor is used to heat the structure, while the measurement resistor is used to measure the temperature and acts as the feedback device for PID control. The temperature of the whole structure depends on the rate of heat production and heat dissipation, and the heat production depends on Joule’s law of the heating resistor. The modes of heat dissipation include heat conduction, heat convection, and heat radiation [27]. According to a previous study [6], heat convection plays a major role in heat dissipation, which is defined by Newton’s law [28], as shown in the following equation:

(6) $$\Phi_{convection}=hA\Delta T,$$

where *Φ_convection_* is the rate of heat convection, *h* is the natural convective heat transfer coefficient, *A* is the area through which heat flux passes, and ∆*T* is the temperature difference between the chip and environment.

During heating, part of the heat will be absorbed by the structure and the rest will be dissipated mainly by thermal convection. The difference between the absorbed heat and the heat emitted by the accelerometer to the outside per unit time is the rate of change of the accelerometer heat storage, as shown in the following equation:

(7) $$Q-hA\left(T-T_{a}\right)=C\frac{dT}{dt},$$

where *Q* is the heat generated by the heating resistor, *T* is the temperature of the structure, *T_a_* is the ambient temperature, which we consider to not produce a change, and *C* is the specific heat capacity. Converting this into incremental form and performing the Laplace transform, we can obtain the transfer function of the heating resistance as:

(8) $$G_{H}\left(s\right)=\frac{1}{Cs+hA}=\frac{K_{1}}{T_{1}s+1},$$

where *T*_1_ is the time constant, which is numerically equal to *C*/*hA*, and *K*_1_ is the gain coefficient, which is numerically equal to 1/*hA*.

Similarly, the measurement resistor has first-order inertia for the temperature-to-resistance conversion, making the entire controlled plant an approximate second-order inertial element with the transfer function:

(9) $$G\left(s\right)=\frac{T\left(s\right)}{Q\left(s\right)}=\frac{K}{\left(T_{1}s+1\right)\left(T_{2}s+1\right)}.$$

By performing a z-transform with the zero-order holder and writing in the time-domain form, we can obtain the following equation:

(10) $$y\left(t\right)=-a_{1}y\left(t-1\right)-a_{2}y\left(t-2\right)+b_{1}u(t-1)+b_{2}u(t-2),$$

where *a*_1_, *a*_2_, *b*_1_, and *b*_2_ are the parameters of the transfer function. Equation (10) gives the relationship between controller output *u* and open-loop output *y*, while *a*_1_, *a*_2_, *b*_1_, and *b*_2_ are unknown parameters, which should be identified by measuring the response of the open-loop output.

**Figure 4:**
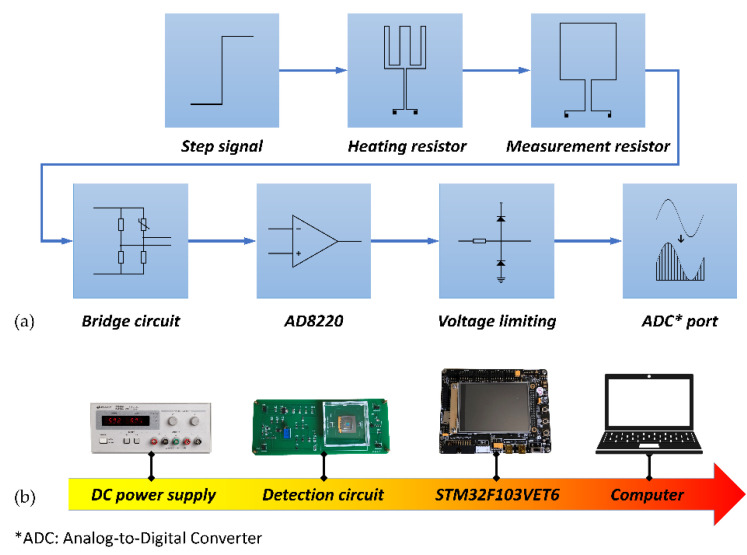
System identification experiment. (**a**) Flow chart for identifying the transfer function of the controlled plant; (**b**) hardware flow of the experiment.

**Figure 5:**
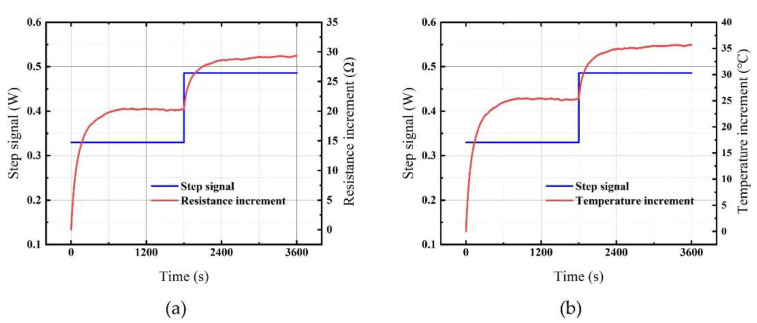
Step response curves. (**a**) Resistance increment; (**b**) temperature increment.

The flow chart of the step response experiment is shown in Figure 4a, and the system diagram of the experiment is shown in Figure 4b. A step signal with a power of 0.33 W (15 V/0.022 A) was input through a voltage source to the heating resistor at *t* = 0 s. A four-arm bridge circuit was utilized to detect the resistance of the measurement resistor. The sampling rate was *f* = 20 Hz. After 1800 s, the response curve reached a steady-state, and a step signal with an alternative power of 0.486 W (18 V/0.027 A) was input until the response curve reached a steady-state again. The step signal of the input and the step response curve of the output are depicted in Figure 5. We wrote the algorithm using the least-squares method to identify *a*_1_, *a*_2_, *b*_1_, and *b*_2_, and the transfer function of the controlled plant was determined as shown in the following equation.

(11) $$G\left(z\right)=\frac{0.08592z^{-1}-0.05117z^{-2}}{1-0.9313z^{-1}-0.06825z^{-2}}.$$

### 3.3. Adaptive Adjustment

PID control is tractable and has been used extensively in practice, but it lacks real-time tuning and anti-interference ability because the parameters remain constant during the control process. For the microaccelerometer chip, it is necessary to make the temperature control flexible to adjust for environmental changes and output deviations of the controller. In this paper, we combine fuzzy logic control [29] with normal PID control to make it more informative. As shown in Figure 3, the fuzzy PID has two inputs including the error and the change of error, which enable the output PID control parameters to adaptively adjust in real time and thus improve system flexibility.

Fuzzy PID control has the following steps:

1. Fuzzification. Scale the input quantities *e*(*t*) and *ec*(*t*) and then determine their fuzzy linguistic values and corresponding affiliation functions.
2. Fuzzy rules. Create a fuzzy rule base. For example, ‘if *e* is A_1_ and *ec* is A_2_, then Δ*K_p_* is A_3_ and Δ*K_i_* is A_4_ and Δ*K_d_* is A_5′_, where A_1_, A_2_, A_3_, A_4_, and A_5_ are linguistic values.
3. Fuzzy inference. Reasoning decisions based on input values and fuzzy rules.
4. Defuzzification. Convert the control quantity obtained by inference into the control output.

Regarding the temperature control method of the microaccelerometer chip proposed in this paper, *e*(*t*) was obtained by making a difference between the feedback of the measurement resistor and the target value, and *ec*(*t*) was the difference of two consecutive errors. In general, the values of *e*(*t*) and *ec*(*t*) are within –1 to 1 times the initial error. We took *e* and *ec* as the input language variables and Δ*K_p_*, Δ*K_i_*, Δ*K_d_* as the output language variables of the fuzzy controller. These five variables were then transformed into seven linguistic values: NB, NM, NS, ZO, PS, PM, and PB, which represented negative big, negative medium, negative small, zero, positive small, positive medium, and positive big, respectively. The default range of these variables was (–6, 6) and in practice would be multiplied by a scale factor. This step was called fuzzification, and we defined the affiliation functions of NB and PB as Gaussian type, while the affiliation functions of other five linguistic values as triangle type. The affiliation function and fuzzy logic were utilized to obtain a set of outputs [Δ*K_p_*, Δ*K_i_*, Δ*K_d_*] from each input of *e* and *ec*.

In incremental PID, *K_p_* acted as *ec*(*t*), *K_i_* acted as *e*(*t*), *K_d_* acted as the change of *ec*(*t*), and the design of fuzzy rules required many experiments and adjustments. The fuzzy control rules of Δ*K_p_*, Δ*K_i_*, and Δ*K_d_* are shown in Table 1, Table 2 and Table 3. The designated fuzzy rules are as follows: when |*e*| is large, *K_i_* and *K_d_* increase, and *K_p_* decreases to speed up the response and reduce the impact of *ec*; when |*e*| is small, *K_i_* increases and *K_d_* decreases to keep the system stable; and when *ec* is negative, a smaller *K_i_* should be taken to prevent overshoot. Meanwhile, to prevent the output curve from diverging, *K_p_* should be as small as appropriate when |*ec*| is positive and large.

**Figure 6:**
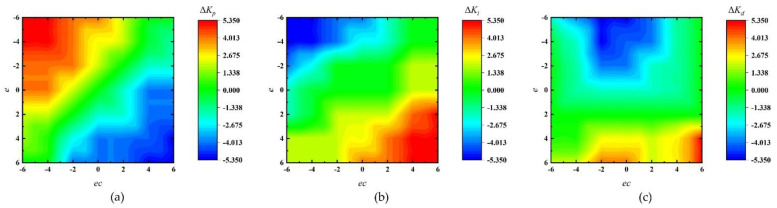
Output surfaces. (**a**) Proportion coefficient; (**b**) integral coefficient; (**c**) differential coefficient.

According to the input values and fuzzy control rules, the relevant parameters were set to obtain the output surfaces of Δ*K_p_*, Δ*K_i_*, and Δ*K_d_* in the theoretical domain, as shown in Figure 6. Here, we used the centroid method for defuzzification, which had smoother output inference control. In the actual control, a pair of *e*(*t*) and *ec*(*t*) was obtained at each moment and then divided by a certain scaling factor. A set of current control variables were obtained from the output surfaces and then scaled to obtain PID control increments [Δ*K_p_*, Δ*K_i_*, Δ*K_d_*] that were added to the initial PID control parameters. The scale factor of the error and change of the error were set to 25/6 as per experiments and comparisons, and the scale factors of Δ*K_p_*, Δ*K_i_*, Δ*K_d_* were 0.33, 0.10, and 0.01, respectively. We believe that the use of fuzzy control can adaptively adjust the control parameters over time to improve the control performance.

### 3.4. Optimization of PID Control Parameters

The adjustment of PID control parameters is a very important part of PID control. Commonly used pieced-together methods and critical proportionality methods rely on experience and observation, resulting in poor control accuracy. Regarding the investigated temperature control method, we mainly focused on parameters such as rise time, settling time, maximum overshoot, and steady-state error, wherein the symbol and meaning of parameters are outlined in Table 4. To gain a short rise time, short settling time, small maximum overshoot, and small steady-state error, we optimized the PID control parameters by using a genetic algorithm [30].

The genetic algorithm performs natural selection according to the fitness function, which judges the degree of excellence of the parameter set. The aforementioned requirements of short rise time, short settling time, small maximum overshoot, and small steady-state error were translated into the fitness function, which could be measured by adding the integral of these parameters by certain weights. Therefore, the final time-varying fitness function has the following form:

(12) $$fitness=\int \left(w_{1}\cdot \left|e\left(t\right)\right|+w_{2}\cdot {\left(\Delta u\left(t\right)\right)}^{2}+w_{3}\cdot e_{over}\left(t\right)\right)dt+w_{4}\cdot t_{rise},$$

where *fitness* is the value of fitness function, *e*(*t*) is the error, Δ*u*(*t*) is the change value of the controller output, *e_over_*(*t*) is the overshoot, *t_rise_* is the rise time, *w*_1_ = 0.002, *w*_2_ = 0.01, *w*_3_ = 100, and *w*_4_ = 1 are the weight values.

**Figure 7:**
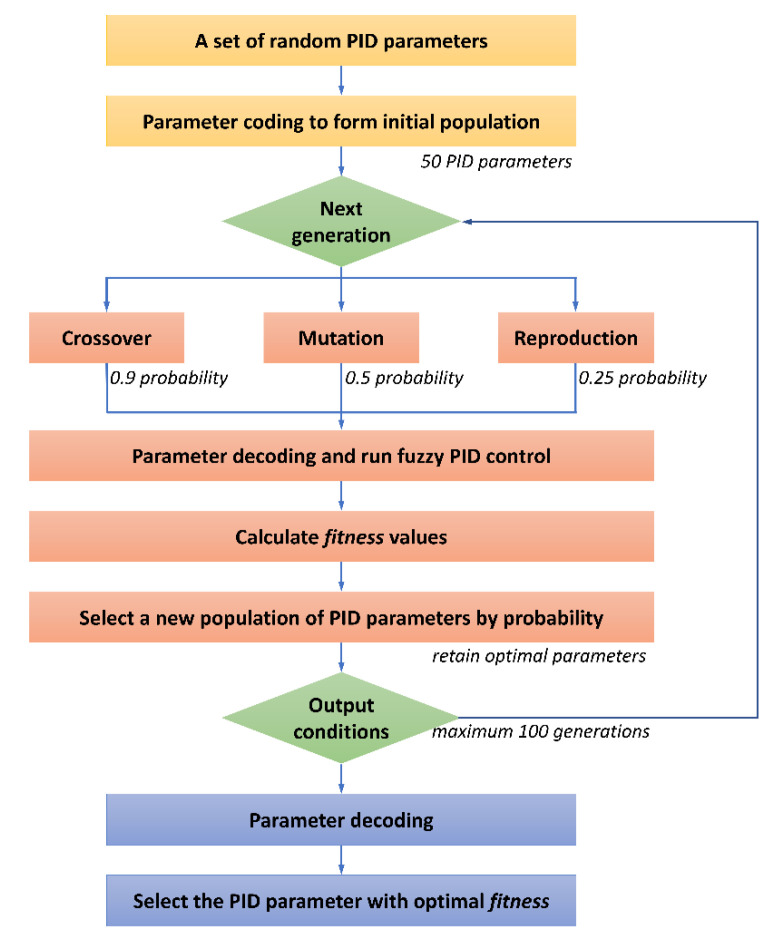
Genetic algorithm optimization process for PID control parameters.

The flow chart of genetic algorithm optimization for PID control parameters is shown in Figure 7. First, a set of PID control parameters within the given upper and lower limits were randomly generated, and a floating-point code was used to form the initial population with a size of 50. Second, generational cycles were done to perform crossover, mutation, and reproduction with probabilities of 0.9, 0.5, and 0.25, respectively. Then, the fitness values were calculated after running fuzzy PID control and decoding the parameters of each generation. Parameters were arranged in ascending order according to the fitness values and selected according to a certain survival probability, and 50 PID parameters of this generation were retained. In the selection process, the optimal combination of parameters for each generation was retained to avoid degradation. The output parameters were decoded and the parameter corresponding to the minimum *fitness* was taken as the initial control parameter of the fuzzy PID. The optimal PID control parameters can be obtained by using the genetic algorithm.

## 4. Results and Discussion

The target of the microaccelerometer chip’s temperature control can be concluded to be stable and fast. More specifically, stable denotes that the temperature oscillation is smaller than 0.01 °C, and fast means a shorter rise time. It should be pointed out that this control system should further reduce the overshoot because it only has heating hardware and the heat-dissipating method is just natural heat dissipation.

In practice, the initial environmental temperature was set to room temperature, which was 25 °C, and the control target was 50 °C. Note that the temperature distribution of the microaccelerometer chip was usually higher than the ambient temperature in the steady-state case, because the heating and dissipation of the chip were in equilibrium, wherein the heat transfer rate is proportional to the temperature difference between the ambient temperature and the chip boundary. The lower bound of the PID controller output *u*(*t*) was set to 0 W (no heating) and the upper bound was 1 W (full power heating) considering the controller output voltage limit, which was 25 V, and the resistance of the heating resistor, which was 625 ohms. The simulation time was set to 120 s first, and the sampling rate was 20 Hz.

We performed three temperature control methods as follows:

1. Normal PID. A suitable set of PID parameters was determined by the trial-and-error method, and the parameters were *K_p_*_0_ = 2.50, *K_i_*_0_ = 0.20, and *K_d_*_0_ = 0.25;
2. Fuzzy PID. A fuzzy strategy was utilized to modify the normal PID, as mentioned in Section 3.3, while the control variables remained positive;
3. Optimized fuzzy PID. The initial parameters of fuzzy PID were optimized by the genetic algorithm mentioned in Section 3.4. The final *fitness* was 63.2715 after several iterations to reduce the randomness, and the parameters were *K_p_*_0_ = 2.2009, *K_i_*_0_ = 0.7999, and *K_d_*_0_ = 0.0105.

**Figure 8:**
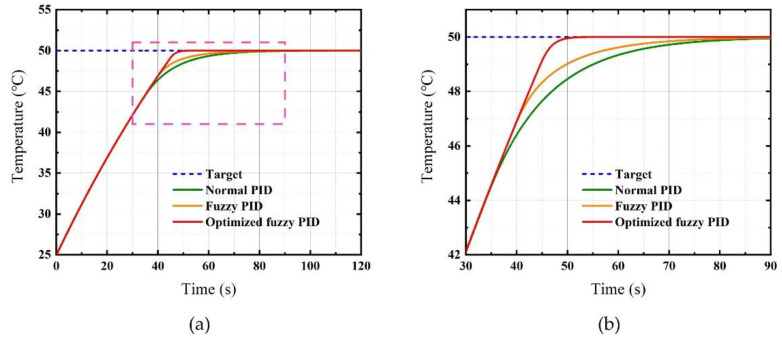
Temperature control performance. (**a**) Comparison of applying three methods; (**b**) red dashed box part of (**a**) enlarged.

The simulation results of the three methods are compared and represented in Figure 8, while the control indexes are listed in Table 5. Figure 8b is an enlarged partial view of Figure 8a, marked with a red dashed box. It is obvious that the fuzzy PID has a faster response and goes into the steady-state earlier than the normal PID, with a rise time reduction of 2.6 s and a settling time reduction of 7.15 s, respectively. In addition, the maximum overshoot of fuzzy PID is −0.00254 °C, which is better than normal PID. The steady-state error decreases by 0.00146 °C and the *fitness* decreases by 3.2685 when using fuzzy PID. It is clear that the results from using fuzzy PID are significantly better than the results from using the normal PID, but there is still room for improvement.

The results of the optimized fuzzy PID show better performance with a faster response and better steady-state characteristics, which meet our requirements. The rise time and settling time were reduced by 2.95 s and 57.2 s compared to that of the normal PID, improving by 6.65% and 52.16%, respectively. With a maximum overshoot of only 4.19 × 10^−9^ °C (this is a simulation value and does not represent the actual measurement results), the proposed method can precisely obtain the control target of 50 °C and this level of overshoot is well within the acceptable range. The steady-state error after the settling time is 0.000217 ℃, which is 30.69 times smaller than the steady-state error from the normal PID.

**Figure 9:**
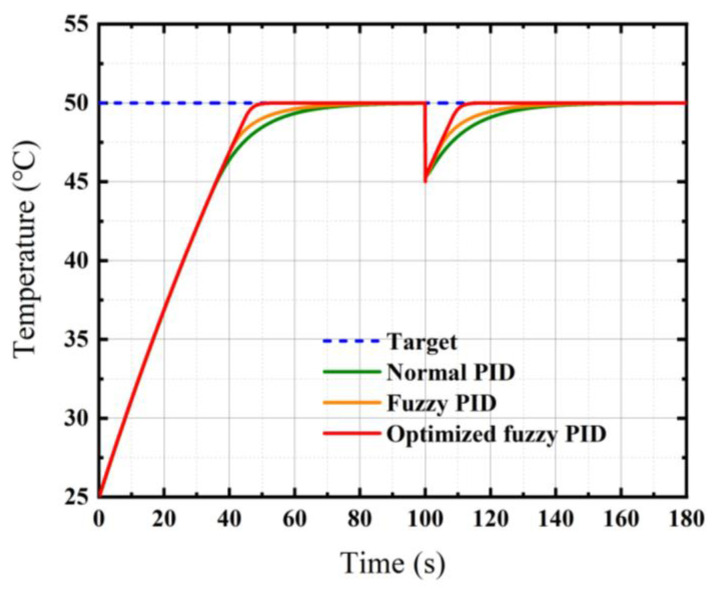
Comparison of temperature control performance while a disturbance was added.

In the actual control process, disturbances may occur due to sudden changes in the external environment or errors in the control system. The control method in this paper allows for a quick response when a disturbance is generated. As shown in Figure 9, the aforementioned three control methods were tested under a disturbance. The simulation time was set to 180 s and a disturbance was added at *t* = 100 s with a temperature drift of 5 °C. We consider this an extreme case because our investigated object is an unpackaged chip, while the chip is packaged for protection and insulation in reality. Typically, the chip of a high-precision microaccelerometer does not operate with a sudden wide range of changes in temperature in terms of the package. In this case, the optimized fuzzy PID has a significantly faster response, and the settling time is listed in Table 6. The method based on the genetic algorithm and fuzzy PID control only takes 15.90 s to return to a steady state, which is much faster than the other two methods. This strongly validates the effectiveness and robustness of the modified temperature control method based on a genetic algorithm and fuzzy PID control.

From the simulation and comparison results, it can be concluded that the normal PID has a good effect, but it has no self-adaptive capability and cannot adjust the parameters in real-time according to the field conditions, which makes the system performance still insufficient. Compared to other methods for microsensors, Li [31], Xia [21], Yang [23], Xia [24], and Jiang [26], their temperature control errors are 0.01 °C, 0.3 °C, 0.067 °C, 0.2 °C, and 0.5 °C respectively. According to the simulation results, using the method proposed in this paper, the temperature control error can decrease by one order of magnitude without compromising the rise time and overshoot, which can be applied to other microsensors. Fuzzy PID makes the self-adaption change possible, improving the dynamic and steady-state characteristics, and has obvious advantages in anti-disturbance and robustness. In addition, using the genetic algorithm for optimization helps to address the issue of empirically adjusting the settings of PID control parameters and fuzzy rules.

## 5. Conclusions

External temperature changes have remained obscure for the practical application of microaccelerometers. This paper proposes a modified PID temperature control method that combines the merits of a fuzzy strategy and a genetic algorithm to enable high-performance and readily adjustable temperature control. This approach is designed for a microaccelerometer chip with a three-layer semiclosed structure, which results in some limitations in temperature control. We first identify the transfer function of the controlled plant by a self-built measurement system. Then, the PID control parameters are optimized by a genetic algorithm in conjunction with a designated fitness function. The parameters are further optimized by a fuzzy strategy to realize real-time responsiveness. Simulation results show that the temperature control method can achieve a settling time reduction of 57.2 s, a very small maximum overshoot, an approximately 30 times improvement in steady-state error, and greater robustness. This method incorporates several key features, such as high performance, strong adaptability, and flexibility, thus making it widely applicable not only to inertial devices but also to other microsensors that have small semiclosed structures.

## Figures and Tables

**Table 1:** Fuzzy control rules of Δ*K_p_*.

*e*	*ec*						
	NB	NM	NS	ZO	PS	PM	PB
**NB**	PB	PB	PM	PM	PS	ZO	ZO
**NM**	PB	PB	PM	PS	PS	ZO	NS
**NS**	PM	PM	PM	PS	ZO	NS	NS
**ZO**	PM	PM	PS	ZO	NS	NM	NM
**PS**	PS	PS	ZO	NS	NS	NM	NM
**PM**	PS	ZO	NS	NM	NM	NM	NB
**PB**	ZO	ZO	NM	NM	NM	NB	NB

**Table 2:** Fuzzy control rules of Δ*K_i_*.

*e*	*ec*						
	NB	NM	NS	ZO	PS	PM	PB
**NB**	NB	NB	NM	NM	NS	ZO	ZO
**NM**	NB	NB	NM	NM	NS	ZO	ZO
**NS**	NM	NS	ZO	ZO	ZO	PS	PS
**ZO**	NS	ZO	ZO	ZO	ZO	PS	PS
**PS**	NS	ZO	PS	PS	PS	PM	PB
**PM**	PS	PS	PS	PS	PM	PB	PB
**PB**	PS	PS	PS	PM	PM	PB	PB

**Table 3:** Fuzzy control rules of Δ*K_d_*.

*e*	*ec*						
	NB	NM	NS	ZO	PS	PM	PB
**NB**	NS	NM	NB	NB	NM	NS	ZO
**NM**	ZO	NS	NB	NM	NM	NS	ZO
**NS**	ZO	NS	NM	NM	NS	NS	ZO
**ZO**	ZO	NS	NS	NS	NS	NS	ZO
**PS**	ZO	ZO	ZO	ZO	ZO	ZO	ZO
**PM**	ZO	ZO	PS	PS	PS	PS	PB
**PB**	PS	PS	PM	PM	PS	PS	PB

**Table 4:** Symbol’s description of PID control performance.

Symbol	Meaning	Description
*t_rise_*	Rise time	Time from start to the first arrival at 90% initial error
*t_settling_*	Settling time	Time from start to stabilization within error of 0.01 °C
*e_maxovershoot_*	Maximum overshoot	The error of the maximum overshoot value of the target value
*e_delta_*	Steady-state error	The average error after *t_settling_*

**Table 5:** Comparison of temperature control results applying three methods.

Control Method	*t_rise_* (s)	*t_settling_* (s)	*e_maxovershoot_* (°C)	*e_delta_* (°C)	*fitness*
Normal PID	44.30	109.65	−0.00416	0.00666	67.5951
Fuzzy PID	41.70	102.50	−0.00254	0.00520	64.3266
Optimized fuzzy PID	41.35	52.450	4.19 × 10^−9^	0.000217	63.2715

**Table 6:** Comparison of settling time for three control methods while a disturbance was added.

Control	Settling Time (s)
Normal PID	73.40
Fuzzy PID	66.20
Optimized fuzzy PID	15.90

## Data Availability

Not applicable.
